# The Long Non-Coding RNA Nostrill Regulates Transcription of Irf7 Through Interaction With NF-κB p65 to Enhance Intestinal Epithelial Defense Against *Cryptosporidium parvum*

**DOI:** 10.3389/fimmu.2022.863957

**Published:** 2022-04-07

**Authors:** Nicholas W. Mathy, Silu Deng, Ai-Yu Gong, Min Li, Yang Wang, Olivia Burleigh, Andrew Kochvar, Erin R. Whiteford, Annemarie Shibata, Xian-Ming Chen

**Affiliations:** ^1^ Department of Medical Microbiology and Immunology, Creighton University School of Medicine, Omaha, NE, United States; ^2^ Department of Microbial Pathogens and Immunity, Rush University Medical Center, Chicago, IL, United States; ^3^ Department of Biology, Creighton University, Omaha, NE, United States; ^4^ Creighton University, School of Medicine, Omaha, NE, United States

**Keywords:** *Cryptosporidium*, intestinal epithelial cells, long non-coding RNA, Nostrill, NR_126553, 2500002B13Rik, IRF7, NF-κB

## Abstract

The cells of the intestinal epithelium establish the frontline for host defense against pathogens in the gastrointestinal tract and play a vital role in the initiation of the immune response. Increasing evidence supports the role of long non-coding RNAs (lncRNAs) as critical regulators of diverse cellular processes, however, their role in antimicrobial host defense is incompletely understood. In this study, we provide evidence that the lncRNA Nostrill is upregulated in the intestinal epithelium following infection by *Cryptosporidium parvum*, a globally prevalent apicomplexan parasite that causes significant diarrheal disease and an important opportunistic pathogen in the immunocompromised and AIDS patients. Induction of Nostrill in infected intestinal epithelial cells was triggered by NF-κB signaling and was observed to enhance epithelial defense by decreasing parasitic infection burden. Nostrill participates in the transcriptional regulation of *C. parvum*-induced Irf7 expression through interactions with NF-κB p65, and induction of Nostrill promotes epigenetic histone modifications and occupancy of RNA polymerase II at the *Irf7* promoter. Our data suggest that the induction of Nostrill promotes antiparasitic defense against *C. parvum* and enhances intestinal epithelial antimicrobial defense through contributions to transcriptional regulation of immune-related genes, such as *Irf7*.

## Introduction

*Cryptosporidium* spp. are an apicomplexan obligate intracellular parasite genus that typically colonize the gastrointestinal tract causing gastroenteritis and diarrheal disease. In immunocompetent adults, Cryptosporidial disease is typically self-limited. However, *Cryptosporidium* remains a significant cause of morbidity and mortality in young children, particularly in developing countries, and in immunocompromised patients where it is recognized as an important opportunistic pathogen in those afflicted with AIDS (1). Despite the global disease burden caused by *Cryptosporidium* spp. there is no fully effective treatment available (2). The most frequently identified species in human infections are *C. parvum* and *C. hominis* (2). *C. parvum* is of particular concern due to its potential for zoonotic transmission, while the host range of *C. hominis* is limited to humans and primates (3). Upon infection, *Cryptosporidium* spp. attach to the apical membrane of host epithelial cells and are internalized in a parasitophorous vacuole, a unique intracellular but extracytoplasmic structure, where the parasite persists during its life cycle (4). Due to the “minimally invasive” nature of the infection in the intestinal epithelium, the intestinal epithelial cells play a central role in the initiation of the immune response against *Cryptosporidium*.

Gastrointestinal epithelial cells are critical mediators of mucosal immunity in response to luminal pathogens and play an important role in crosstalk between the lumen and the underlying mucosa (5, 6). The cells of the intestinal epithelium contribute to the mucosal barrier to protect hosts from infection through mucus secretion, antimicrobial peptide production, and contributions to tight junction integrity (7). Upon infection, cells of the intestinal epithelium respond through innate immunity mechanisms to release cytokines and chemokines which direct immune effector cells to the source of infection (8, 9). Signaling pathways, such as TLR4-mediated NF-κB activation, play an important role in clearance of *C. parvum* from infected epithelial cells (10, 11). Previous studies have reported the importance of type II interferon at the site of infection for anti-Cryptosporidial defense (12). Recent evidence also demonstrates induction of a strong type I interferon response in *Cryptosporidium* infected epithelial cells and that deficiency in type I interferons results in a higher parasite burden (13). Increased understanding of the regulation of the interferon response to *C. parvum* infection may provide new insights for enhanced therapeutic strategies.

Long non-coding RNAs (lncRNAs) are RNA molecules greater than 200 nucleotides in length that typically lack coding potential. LncRNAs have been identified that perform critical roles in cellular processes such as cell cycle regulation, tumorigenesis, apoptosis, and immune surveillance (14). Studies have shown that lncRNAs can regulate target gene expression through their ability to interact with DNA, RNA, and proteins. Molecular mechanisms of gene regulation by lncRNAs include the “guide” or “scaffold” whereby lncRNAs interact with RNA-binding proteins including transcription factors or histone-modifying enzymes, or with larger molecular complexes, and direct them to specific genomic loci to impact target gene expression (15). Numerous lncRNAs have been previously identified that contribute to the intestinal epithelial inflammatory response and anti-Cryptosporidial defense (16–18). For example, NR_045064 was observed to regulate the expression of inducible nitric oxide synthase (iNOS) and granulocyte-macrophage colony-stimulating factor (GM-CSF) in intestinal epithelial cells in response to *C. parvum* infection through epigenetic modifications by the p300/WDR5/MLL complex, while a different lncRNA, XR_001779380, interacts with a Stat1/SWI/SNF chromatin remodeling complex to enhance IFN-γ-mediated inflammatory gene transcription (17, 18).

In this study, we investigated the role of the lncRNA Nostrill, which we previously identified in the regulation of iNOS expression in microglia (19), in anti-*C. parvum* defense using murine models of intestinal cryptosporidiosis. We observed significant induction of Nostrill after *C. parvum* infection *in vitro*, *ex vivo* using a 2D enteroid monolayer, and *in vivo*. Manipulation of Nostrill expression in intestinal epithelial cells revealed a significant impact on *C. parvum* parasite burden. We propose that the underlying molecular mechanism of Nostrill’s contribution to anti-*C. parvum* defense is through transcriptional regulation of Irf7 through interactions with NF-κB p65. Our data support that Nostrill may facilitate intestinal epithelial defense against *C. parvum* through regulation of the interferon response.

## Materials and Methods

### Cell Lines, Enteroids, and Animals

The neonatal intestinal epithelial cell line (IEC4.1) was a kind gift from Dr. Pingchang Yang (McMaster University, Hamilton, Canada). DMEM-F-12 culture media was supplemented with 10% fetal bovine serum (FBS) (Ambion, Austin, TX) and 1% penicillin/streptomycin (ThermoFisher Scientific, Waltham, MA). HCT-8 cells were purchased from ATCC (Manassas, VA). RPMI-1640 cell culture media was supplemented with 10% fetal bovine serum (FBS) (Ambion, Austin, TX) and 1% penicillin/streptomycin (ThermoFisher Scientific, Waltham, MA). Cells were grown in tissue culture flasks at 37°C in 5% CO_2_ and allowed to reach 80% confluency before passage.

C57BL/6J mice were originally purchased from the Jackson Laboratory. Mice at the age of 5 days after birth were used for *in vivo* infection. Intestinal villus/crypt components from mice at 5 days after birth were isolated and cultured as described in our previous studies (20). Briefly, small intestines were opened longitudinally and washed with ice-cold Ca^2+^ and Mg^2+^ free PBS, then were cut into 1–2 mm fragments and washed with ice-cold Ca^2+^ and Mg^2+^ free PBS 3 times. The cut fragments were incubated in ice-cold 2 mM PBS/EDTA at 4°C for 30 min with gentle rotation followed by vigorous shake until the PBS solution was mostly opaque with dislodged crypt and villus particles. Large tissue fragments were removed through a 100-μm cell strainer (Becton-Dickinson Bioscience, Franklin Lakes, NJ). The pass through was centrifuged 150g for 5 min at 4°C and the pellet was collected as the intestinal epithelium. 2D monolayers derived from 3D enteroids were cultured as previously described (20, 21).

### *C. parvum* Oocysts

*C. parvum* oocysts harvested from calves inoculated with the Iowa strain originally obtained from Harley Moon at the National Animal Disease Center (Ames, IA) were purchased from a commercial source (Bunch Grass Farms, Deary, ID). Oocysts were purified using a modified ether extraction technique and then suspended in phosphate-buffered saline (PBS) and stored at 4°C. For *in vitro*, 2D monolayer *ex vivo*, and *in vivo* infection, oocysts were treated with 1% sodium hypochlorite on ice for 20 min and washed 3 times with Dulbecco’s modified Eagle medium (DMEM) culture media. All parasite preparations were tested using the Limulus amebocyte lysate gel formation test as previously described to exclude the possibility of contamination with lipopolysaccharides (22, 23).

### Infection Models and Assays

For *in vitro* infection using IEC4.1 and HCT-8 cells, or *ex vivo* infection using 2D monolayers derived from 3D enteroids, infection was performed in culture medium containing viable *C. parvum* oocysts after treatment with 1% sodium hypochlorite (1:1 ratio of oocyst:cell). Cells were then cultured for 4 h at 37°C for attachment and invasion by the parasites. Cells were then washed with cell medium three times to remove free parasites. Cells were cultured for additional time periods as described.

The neonatal murine infection model of intestinal cryptosporidiosis was used for *in vivo* experiments (24–26). Neonates (5 days after birth) received *C. parvum* oocysts by oral gavage (10^5^ oocysts per mouse) to develop intestinal cryptosporidiosis. Control mice received an equal volume of PBS by oral gavage. At 24, 48, and 72 h after *C. parvum* oocysts or PBS administration, at least five animals were sacrificed per condition, and ileal intestinal tissue was collected for biochemical analyses.

### Quantitative Real-Time PCR (qRT-PCR) Analysis

For real-time PCR analysis, total RNA was isolated from cells with Trizol reagent (Applied Biosystems, Carlsbad, CA). An amount of 200 ng total RNA was reverse-transcribed using the iScript Reverse Transcription Supermix (Bio-Rad, Hercules, CA). Comparative real-time PCR was performed using the SYBR Green PCR Master Mix (Applied Biosystems, Carlsbad, CA) on the Bio-Rad CFX96 Touch™ Real-Time PCR Detection Systemn (Bio-Rad, Hercules, CA). The sequences for all the primers are listed in **Supplementary Table 1**. Normalization was performed using Gapdh. Relative expression was calculated using the comparative Ct (ΔΔCt) method.

### siRNAs and Plasmids

For gene silencing, the small interfering RNA (siRNA) duplexes for mouse Nostrill were synthesized using Integrated DNA Technologies. The siRNA sequences are listed in **Supplementary Table 1**. Cells were treated with siRNAs (final concentration, 60 nM) using Lipofectamine RNAiMAX (Invitrogen, Carlsbad, CA) according to the manufacturer’s instructions. For Nostrill overexpression, Nostrill cDNA was amplified through PCR, inserted into the PTarget (Promega, Madison, WI) expression vector to generate PTarget-Nostrill, and subsequently sequenced. According to the manufacturer’s protocol, cells were transfected with plasmid DNA using Lipofectamine 2000 (Invitrogen, Carlsbad, CA). Quantitative RT-PCR was used to determine the significant alteration of each target gene.

### RNA Immunoprecipitation (RIP) Assay

Formaldehyde crosslinking RIP was performed as described (27). Briefly, lysates were precleaned with 20 μl of PBS washed Magna ChIP Protein A + G Magnetic Beads (Millipore, Burlington, MA). The precleaned lysate (250 μl) was then diluted with the whole cell extract buffer (250 μl), mixed with the specific antibody-coated beads, and incubated with rotation at 4 °C for 4 h, followed by 4 times washing with the whole cell extract buffer containing protease and RNase inhibitors. The collected immunoprecipitated RNP complexes and input were digested in RNA PK Buffer pH 7.0 (100 mM NaCl, 10 mM TrisCl pH 7.0,1 mM EDTA, 0.5% SDS) with addition of 10 μg Proteinase K and incubated at 50°C for 45 min with end-to-end shaking at 400 rpm. Formaldehyde cross-links were reversed by incubation at 65°C with rotation for 4 h. RNA was extracted from these samples using Trizol according to the manufacturer’s protocol (Invitrogen, Carlsbad, CA) and treated with DNA-free DNase Treatment & Removal I kit according to the manufacturer’s protocol (Ambion, Austin, TX). The presence of RNA was measured by qRT-PCR as described above. Gene-specific PCR primer pairs are listed in **Supplementary Table 1**. The following antibodies were used for RIP analysis: anti-NF-κB p50 (Santa Cruz, Dallas, TX), anti-NF-κB p65 (Santa Cruz, Dallas, TX), normal mouse IgG (Santa Cruz, Dallas, TX).

### Chromatin Immunoprecipitation (ChIP) Assay

ChIP assays were performed as described previously (28, 29). Briefly, cells were fixed with 1% formaldehyde for 10 min, collected in ice-cold PBS, and resuspended in an SDS lysis buffer. Genomic DNA was then sheared to lengths ranging from 200 to 1000 bp by sonication. Cell extracts were incubated with either anti-NF-κB p65 (Santa Cruz, Dallas, TX), anti-H3K4me3 (Cell Signaling Technology, Danvers, MA), anti-RNA Polymerase 2 (Millipore, Burlington, MA), or normal mouse IgG (Santa Cruz, Dallas, TX) overnight at 4°C, followed by precipitation with protein G-agarose beads. The immunoprecipitates were sequentially washed once with a low-salt buffer, once with a high-salt buffer, once with an LiCl buffer, and twice with a Tris buffer. The DNA–protein complex was eluted, and proteins were then digested with proteinase K for 1 h at 45°C. The DNA was detected by qRT-PCR analysis as described above and analyzed using the fold enrichment method. Gene-specific PCR primer pairs are listed in **Supplementary Table 1**.

### Chromatin Isolation by RNA Purification (ChIRP) Assay

ChIRP analysis was performed as previously reported (19, 27). Briefly, a pool of tiling oligonucleotide probes with affinity specific to the Nostrill sequence was used and glutaraldehyde cross-linked for chromatin isolation. The sequences for each probe are listed in **Supplementary Table 1**; probe 1, 3, 5, and 7 are mixed as the probe pool Odd and probe 2, 4, 6, and 8 as the probe pool Even. The DNA sequences of the chromatin isolates were confirmed and quantified by qRT-PCR using the same primer sets covering the gene promoter regions of interest as for ChIP analysis. A pool of oligo probes for LacZ were served as controls. The percent input method was used to normalize the ChIRP data.

### Statistical Analysis

Data are expressed as mean values and error bars represent standard error of the mean (SEM). Student T test with Bonferroni’s correction or one-way ANOVA followed by Tukey-Kramer *post hoc* tests were performed where appropriate. For determination of significant differences between percents and for multiple comparisons between culture conditions, two-way ANOVA followed by Tukey-Kramer multiple analyses *post hoc* tests were used. p values < 0.05 were considered statistically significant.

## Results

### Upregulation of the lncRNA Nostrill Following *C. parvum* Infection

**Figure 1:**
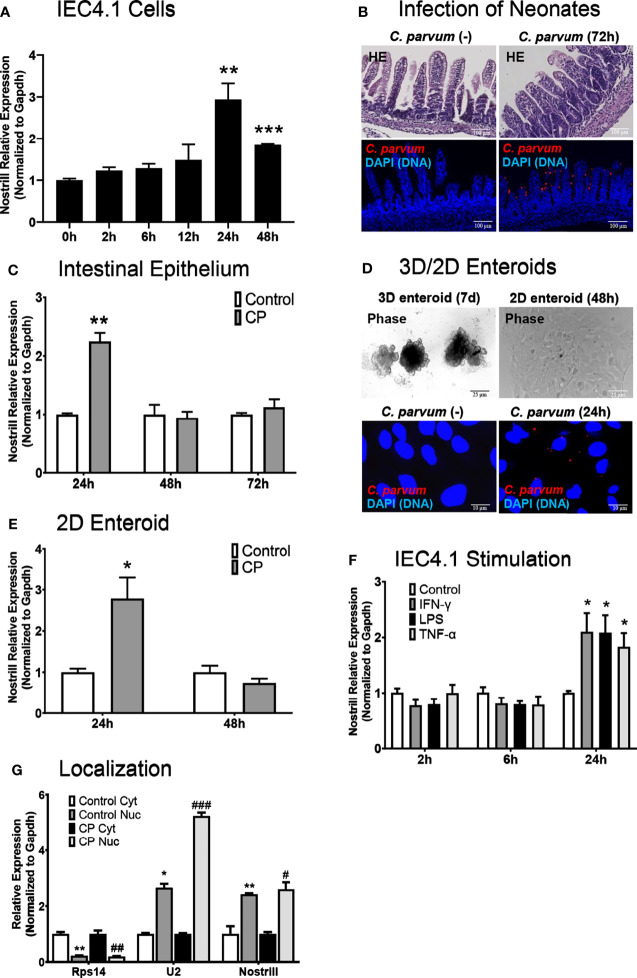
Characterization of Nostrill induction in the intestinal epithelium in response to *C*. *parvum* infection. **(A)** Induction of the lncRNA Nostrill in response to *C. parvum* infection in cultured intestinal epithelial cells. IEC4.1 cells were infected with *C*. *parvum* for 2-48 h and Nostrill induction was measured *via* qRT-PCR. **(B)** *In vivo C*. *parvum* infection of intestinal epithelium in neonatal mice. Neonatal mice at 5 days old received *C*. *parvum via* oral gavage, control mice received PBS. H&E staining shows villous atrophy and crypt hyperplasia. Indirect immunofluorescent staining of the ileum demonstrates *C*. *parvum* infection (red). Scale bar 100 µm. **(C)** Time-course of Nostrill induction in response to *C*. *parvum* infection of the intestinal epithelium *in vivo*. The intestinal epithelium was isolated at 24, 48, and 72 h post-infection and Nostrill expression was quantified by qRT-PCR. **(D)** *Ex vivo C*. *parvum* infection of 2D intestinal epithelial monolayers. The crypt units of the intestinal epithelium were isolated and cultured into 3D enteroids (7 d) and cultured into 2D monolayers, followed by *C. parvum* infection for 24 h Phase contrast images of 3D/2D enteroids are shown, scale bar 25 µm, and immunofluorescent microscopy demonstrate *C. parvum* infection (red), scale bar 10 µm. **(E)** Upregulation of Nostrill in response to *C*. *parvum ex vivo* in 2D intestinal epithelial monolayer. Expression of Nostrill in 2D intestinal epithelial monolayers was measured *via* qRT-PCR. **(F)** Temporal induction of Nostrill in intestinal epithelial cells in response to inflammatory stimuli. IEC4.1 cells stimulated with IFN-γ (20 ng/ml), LPS (10 μg/ml), or TNF-α (20 ng/ml) for 2, 6, or 24 h. Nostrill expression was quantified by qRT-PCR. Data represent means ± SEM from three independent experiments. Gapdh was used as a reference gene for normalization. *p < 0.05, **p < 0.01, and ***p < 0.001 vs control. **(G)** Intracellular localization of Nostrill in response to *C. parvum* infection. IEC4.1 cells were infected with *C. parvum* for 24 h. Nuclear and cytoplasmic fractions were isolated as previously described (32). Rps14, U2, and Nostrill expression was quantified *via* qRT-PCR. Data represent means ± SEM from three independent experiments. *p < 0.05 and **p < 0.01 vs control cytoplasmic fraction. ^#^p < 0.05, ^##^p < 0.01, and ^###^p < 0.001 vs *C*. *parvum* cytoplasmic fraction.

We have previously reported genome-wide RNA transcriptome analysis of *C. parvum*-infected IEC4.1 cells, a transformed but non-tumorigenic neonatal mouse intestinal epithelial cell line (30). Nostrill was additionally identified as being strongly upregulated during genome-wide RNA transcriptome analysis of murine microglia stimulated with LPS, and we have published data on the role of Nostrill in regulating the expression of iNOS in the microglial inflammatory response (19). In response to *C. parvum* infection, many lncRNAs displayed differential expression as previously identified by microarray analysis, including Nostrill (2.47-fold) (17). Nostrill, or NR_126553, is transcribed from the *2500002B13Rik* gene locus on chromosome 8 between the *Sap30* and *Hmgb2* gene loci (**Supplementary Figure 1**) (31). Using qRT-PCR, we confirmed the upregulation of Nostrill in IEC4.1 cells 24 h following *C. parvum* infection at 2.94-fold, p < 0.01 compared to uninfected controls (**Figure 1A**). Nostrill did not display upregulation prior to the 24 h timepoint but remained significantly elevated 48 h post-infection, albeit at a lower level than the 24 h timepoint (**Figure 1A**). To verify biological relevance of Nostrill induction in *C. parvum* infection *in vivo*, we utilized a neonatal murine model of intestinal cryptosporidiosis *via* oral administration of the parasite (**Figure 1B**) to examine expression of Nostrill in the intestinal epithelium (24–26). Nostrill was significantly upregulated 24 h post-infection in the intestinal epithelium, however, its expression returned to basal levels by the 48 h timepoint (**Figure 1C**). An *ex vivo* infection model employing 2D enteroid monolayers derived 3D enteroids originating from the neonatal mouse ileum (**Figure 1D**) was utilized to ensure the significant upregulation of Nostrill observed *in vivo* was due to differential expression in the cells of the intestinal epithelium. Infection of 2D enteroid monolayers does not require microinjection of *C. parvum* as infection of 3D enteroids does (33). *C. parvum* infection of 2D epithelial monolayers displayed a 2.78-fold significant increase in Nostrill expression at 24 h post-infection (**Figure 1E**), consistent with *in vitro* and *in vivo* results (20, 21). We then investigated the response of Nostrill in IEC4.1 cells to known inflammatory mediators, including IFN-γ, LPS, and TNF-α (**Figure 1F**). Nostrill displayed significant upregulation in response to IFN-γ, LPS, and TNF-α at 24 h post-stimulation in response to all three stimuli, providing additional evidence that Nostrill may function broadly in the inflammatory response in different cell types and in response to numerous stimuli (**Figure 1F**) (19). No significant differential expression of Nostrill was observed at earlier timepoints (**Figure 1F**). Initial characterization of the intracellular localization of Nostrill was performed using nuclear and cytoplasmic extraction with the nuclear marker U2 and the cytoplasmic marker Rps14 (**Figure 1G**). Utilizing this technique, Nostrill displayed a preference for nuclear localization and its localization did not change significantly in *C. parvum*-infected cells compared to uninfected controls (**Figure 1G**). Together, this data demonstrates that the lncRNA Nostrill is induced in response to *C. parvum* infection and more broadly in response to stimulation with inflammatory mediators and demonstrates a preference for nuclear localization intracellularly.

### *C. parvum*-Induced Nostrill Upregulation and Antiparasitic Defense Are NF-κB Dependent

**Figure 2:**
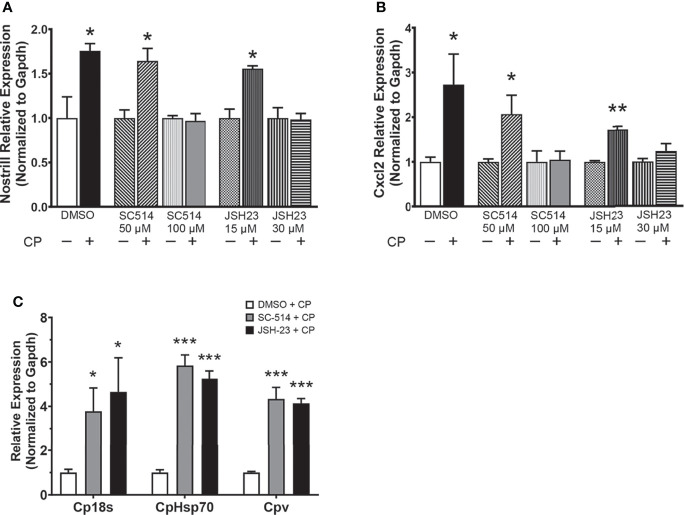
The NF-κB signaling pathway controls *C. parvum*-induced Nostrill expression and parasite infection burden. **(A)** *C*. *parvum*-induced Nostrill expression is NF-κB-dependent in intestinal epithelial cells. IEC4.1 cells were pre-treated with the NF-кB inhibitors SC-514 (50 or 100 μM) or JSH-23 (15 or 30 μM) 1 h prior to *C*. *parvum* infection for 24 h. DMSO was used for as a negative control. **(B)** Validation of inhibition of NF-кB signaling in intestinal epithelial cells. Expression of Cxcl2, a NF-кB target gene, was measured after *C*. *parvum* infection of IEC4.1 cells with or without pretreatment with NF-кB inhibitors. **(C)** NF-κB signaling contributes to defense against *C*. *parvum* in IEC4.1 cells. IEC4.1 cells were pre-treated with the NF-кB inhibitors SC-514 (100 μM) or JSH-23 (30 μM) 1 h prior to *C*. *parvum* infection for 24 h. DMSO was used for as a negative control. *C. parvum* infection burden was quantified *via* qRT-PCR. Data represent means ± SEM from three independent experiments. Gapdh was used as a reference gene for normalization. *p < 0.05, **p < 0.01, ***p < 0.001 vs control.

As NF-κB is a master regulator of the inflammatory response and has been previously shown to control the expression of Nostrill in response to inflammatory stimuli (19), we investigated whether inhibition of NF-κB influences the *C. parvum*-mediated induction of Nostrill in intestinal epithelial cells. Two different NF-κB inhibitors, SC-514 and JSH-23, were used to query the dependence of Nostrill expression on NF-κB signaling in a dose-dependent manner. *C. parvum* infection significantly increased Nostrill expression 1.76-fold compared to uninfected controls (**Figure 2A**). Both SC-514 (100 µM) and JSH-23 (30 μM) inhibited significant upregulation of Nostrill in *C. parvum*-infected cells compared to uninfected controls, while lower doses of NF-κB inhibitors failed to inhibit *C. parvum-*induced Nostrill upregulation (**Figure 2A**). Cxcl2, a known NF-κB response gene, served as a positive control to demonstrate effective inhibition of NF-κB signaling (**Figure 2B**) (34). Without NF-κB inhibition, Cxcl2 showed a 2.73-fold significant upregulation after *C. parvum* infection (**Figure 2B**). At higher doses, both SC-514 (100 μM) and JSH-23 (30 μM) successfully inhibited induction of Cxcl2 in response to *C. parvum* infection (**Figure 2B**). Pretreatment with NF-κB inhibitors at lower doses failed to inhibit upregulation of Cxcl2 following *C. parvum* infection (**Figure 2B**). We additionally confirmed the impact of NF-κB signaling on *C. parvum* infection burden as previously reported (11). Pretreatment with the NF-κB inhibitors SC-514 (100 µM) and JSH-23 (30 μM) followed by infection of IEC4.1 cells with *C. parvum* resulted in a significant increase in parasite infection burden as assessed by qRT-PCR (**Figure 2C**). These data suggest that optimal antiparasitic defense and induction of the lncRNA Nostrill are NF-κB dependent in *C. parvum* infection of intestinal epithelial cells.

### Nostrill Impacts *C. parvum* Infection Burden in Intestinal Epithelial Cells

**Figure 3:**
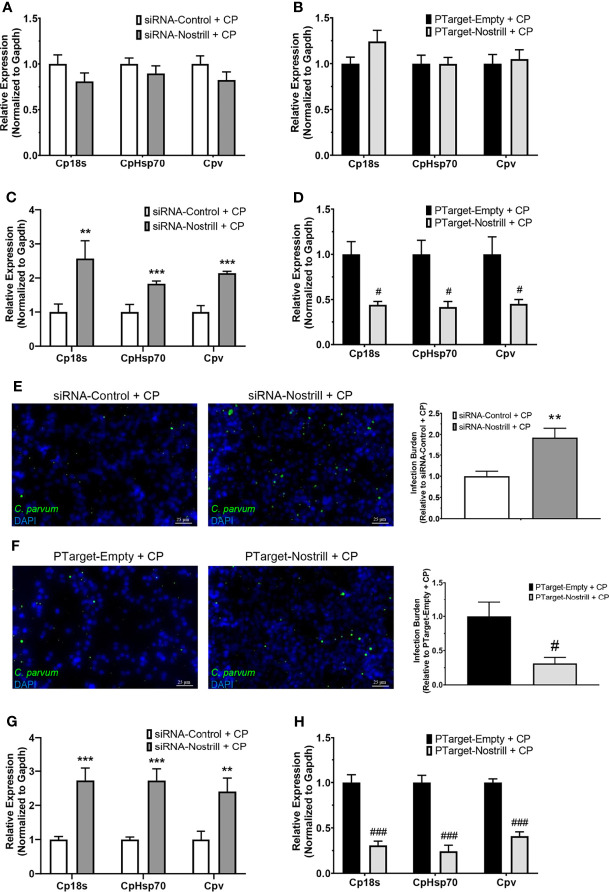
Impact of Nostrill induction on *C. parvum* infection burden in intestinal epithelial cells. **(A)** Impact of Nostrill knockdown on *C*. *parvum* attachment and invasion *in vitro*. IEC4.1 cells were transfected with a control scrambled siRNA (siRNA-Control) or a siRNA specific to Nostrill (siRNA-Nostrill) then infected with *C*. *parvum* for 4 h. *C. parvum* infection burden was quantified *via* qRT-PCR. **(B)** Impact of Nostrill overexpression on *C. parvum* attachment and invasion *in vitro*. IEC4.1 cells were transfected with a Nostrill overexpression plasmid (PTarget-Nostrill) or empty vector control (PTarget-Empty) then infected with *C*. *parvum* for 4 h. *C. parvum* infection burden was quantified *via* qRT-PCR. **(C)** Impact of Nostrill knockdown on *C. parvum* infection *in vitro*. IEC4.1 cells were transfected with a control scrambled siRNA (siRNA-Control) or a siRNA specific to Nostrill (siRNA-Nostrill) then infected with *C*. *parvum* for 24 h *C. parvum* infection burden was quantified *via* qRT-PCR. **(D)** Impact of Nostrill overexpression on *C*. *parvum* infection *in vitro*. IEC4.1 cells were transfected with a Nostrill overexpression plasmid (PTarget-Nostrill) or empty vector control (PTarget-Empty) then infected with *C. parvum* for 24 h. *C. parvum* infection burden was quantified *via* qRT-PCR. **(E)** Impact of Nostrill knockdown on *C. parvum* infection *in vitro*. IEC4.1 cells were transfected with a control scrambled siRNA (siRNA-Control) or a siRNA specific to Nostrill (siRNA-Nostrill) then infected with *C. parvum* for 24 h. *C. parvum* infection burden was quantified *via* indirect immunofluorescence microscopy. DAPI was used to quantify nuclei. Infection burden was quantified as number of *C*. *parvum* (green) present relative to cell number. Scale bar 25 µm. **(F)** Impact of Nostrill overexpression on *C*. *parvum* infection *in vitro*. IEC4.1 cells were transfected with a Nostrill overexpression plasmid (PTarget-Nostrill) or empty vector control (PTarget-Empty) then infected with *C*. *parvum* for 24 h. *C. parvum* infection burden was quantified *via* indirect immunofluorescence microscopy. DAPI was used to quantify nuclei. Infection burden was quantified as number of *C*. *parvum* (green) present relative to cell number. Scale bar 25 µm. **(G)** Impact of Nostrill knockdown on *C*. *parvum* infection burden *ex vivo*. 2D intestinal epithelial monolayers were transfected with a control scrambled siRNA (siRNA-Control) or a siRNA specific to Nostrill (siRNA-Nostrill) then infected with *C*. *parvum* for 24 h. *C. parvum* infection burden was quantified *via* qRT-PCR. **(H)** Impact of Nostrill overexpression on *C*. *parvum* infection *ex vivo*. 2D intestinal epithelial monolayers were transfected with a Nostrill overexpression plasmid (PTarget-Nostrill) or empty vector control (PTarget-Empty) then infected with *C*. *parvum* for 24 h. *C. parvum* infection burden was quantified *via* qRT-PCR. Data represent means ± SEM from three independent experiments. Gapdh was used as a reference gene for normalization. **p < 0.01 and ***p < 0.001 vs siRNA-Control + CP. ^#^p < 0.05 and ^###^p < 0.001 vs PTarget-Empty + CP.

To determine whether Nostrill may contribute to anti-*C. parvum* defense, we infected IEC4.1 cells after knockdown or overexpression of Nostrill then measured the impact on parasite infection burden at different timepoints. The RNAi approach was utilized for Nostrill knockdown and the siRNA targeting Nostrill successfully reduced the level of Nostrill in uninfected and *C. parvum*-infected intestinal epithelial cells compared to a scrambled siRNA (**Supplementary Figure 2**). Complementarily, overexpression was performed *via* transfection with the pTargeT Mammalian Expression Vector containing a cloned insert of Nostrill and resulted in significant induction of Nostrill expression compared to empty vector control in both uninfected and infected cells (**Supplementary Figure 2**). We began by assessing the impact of Nostrill expression on *C. parvum* attachment/invasion into IEC4.1 cells by measuring parasite burden 4 h after infection (35). Neither knockdown nor overexpression of Nostrill significantly affected *C. parvum* infection burden after parasite attachment/invasion (**Figures 3A, B**). We then measured *C. parvum* burden 24 h after infection and found that knockdown of Nostrill significantly increased infection burden compared to scrambled siRNA controls (**Figure 3C**). Conversely, overexpression of Nostrill significantly decreased *C. parvum* burden at 24 h post-infection compared to empty vector controls (**Figure 3D**). Indirect immunofluorescence microscopy was additionally utilized to detect infection burden in IEC4.1 cells. In accordance with results observed by qRT-PCR, knockdown of Nostrill resulted in a significant 1.93-fold increase in *C. parvum* infection burden (**Figure 1E**), while Nostrill overexpression caused a significant 0.31-fold decrease in *C. parvum* infection burden (**Figure 1F**).

We additionally employed the 2D intestinal monolayer model to further examine the impact of Nostrill on *C. parvum* infection burden. Successful knockdown or overexpression of Nostrill was observed (**Supplementary Figure 2**). Similar to the results seen in IEC4.1 cells, knockdown of Nostrill significantly increased the infection burden of *C. parvum* 24 h post-exposure compared to scrambled siRNA controls (**Figure 3G**), while overexpression of Nostrill resulted in a significant decrease in *C. parvum* infection burden compared to empty vector controls (**Figure 3H**). Taken together, the above data suggests that induction of the lncRNA Nostrill contributes to epithelial defense against *C. parvum* infection.

### Impact of Nostrill Induction on Select Defense Genes in Intestinal Epithelial Cells

**Figure 4:**
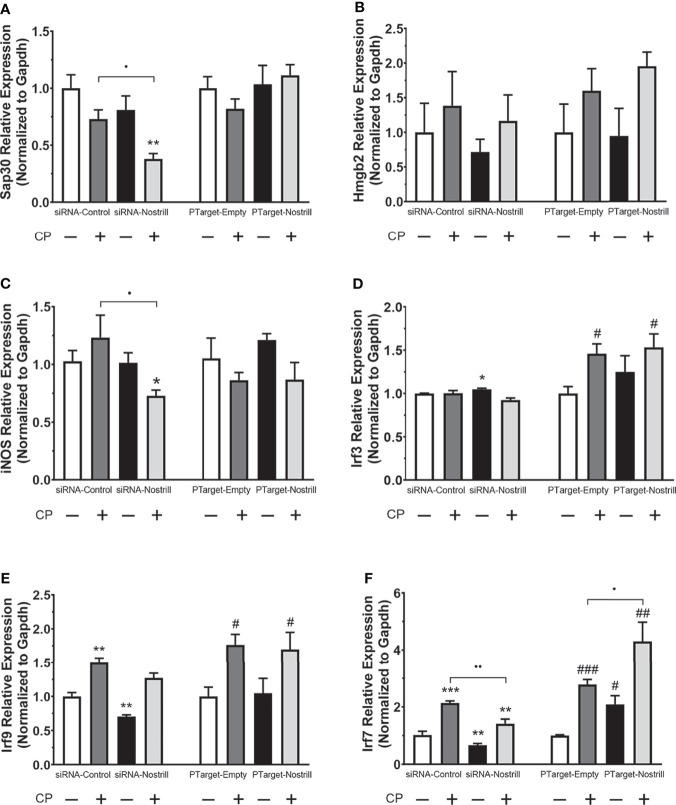
Effect of Nostrill induction on expression of inflammatory genes in intestinal epithelial cells following *C. parvum* infection. For knockdown, IEC4.1 cells were transfected with control scrambled siRNA (siRNA-Control) or a siRNA specific to Nostrill (siRNA-Nostrill). For Nostrill overexpression, IEC4.1 cells were transfected with Nostrill overexpression plasmid (PTarget-Nostrill) or empty vector control (PTarget-Empty). Cells were then infected with*C. parvum* for 24 h. Expression levels of selected inflammatory genes, **(A)** *Sap30*, **(B)** *Hmgb2*, **(C)** *iNOS*, **(D)** *Irf3*, **(E)** *Irf9*, **(F)** *Irf7*, were quantified by using real-time PCR. Gapdh was used as a reference gene for normalization. Data represent means ± SEM from three independent experiments. *p < 0.05, **p < 0.01, and ***p < 0.001 vs uninfected siRNA-Control. ^#^p < 0.05, ^##^p < 0.01, and ^###^p < 0.001 vs uninfected PTarget-Empty. •p < 0.05 and ••p < 0.01 between indicated groups.

Given the significant impact of Nostrill on *C. parvum* infection burden in the intestinal epithelium, we next sought to answer whether this effect may be due to modulation of *C. parvum*-induced defense gene expression by the lncRNA Nostrill. Knowing that lncRNAs have been observed to modulate target gene expression, both *in cis* and *in trans*, we began by evaluating the impact of Nostrill knockdown or overexpression on *C. parvum*-induced expression of the neighboring genes, *Sap30* and *Hmgb2*. *C. parvum* infection in scrambled siRNA controls resulted in a downregulation of Sap30 expression compared to uninfected cells, although this effect was non-significant (**Figure 4A**). Knockdown of Nostrill did not significantly affect Sap30 expression in uninfected cells but caused a significant decrease in Sap30 expression in *C. parvum* infected cells (**Figure 4A**). Overexpression of Nostrill did not significantly impact Sap30 expression (**Figure 4A**). *C. parvum* infection did not significantly affect Hmgb2 expression in the scrambled siRNA or empty vector controls (**Figure 4B**). Neither Nostrill knockdown nor overexpression caused a significant difference in levels of Hmgb2 mRNA (**Figure 4B**). Together, this indicated that Nostrill has little impact on transcriptional regulation *in cis*. We then investigated whether Nostrill impacts the transcription of iNOS as previously observed in microglia (19). Knockdown of Nostrill in *C. parvum* infected cells demonstrated a significant decrease in iNOS expression compared to both uninfected and infected scrambled siRNA controls (**Figure 4C**). Overexpression of Nostrill produced no significant alterations in iNOS expression in intestinal epithelial cells in response to *C. parvum* infection (**Figure 4C**).

Given previous data on the importance of interferon-mediated epithelial anti-*C. parvum* defense (12, 13), we next investigated whether Nostrill may be involved in regulation of the interferon response *via* modulation of interferon regulatory transcription factor (IRF) family members. Induction of Irf3 was not observed following *C. parvum* infection in the scrambled siRNA or Nostrill knockdown samples (**Figure 4D**). However, Nostrill knockdown caused a significant increase in Irf3 expression in uninfected cells compared to scrambled siRNA control. Interestingly, *C. parvum* infection induced Irf3 expression 1.46-fold and 1.54-fold in empty vector and Nostrill overexpression conditions, respectively. The overexpression of Nostrill did not significantly affect Irf3 expression in uninfected cells (**Figure 4D**). Irf9 was significantly induced by *C. parvum* infection to 1.50-fold and 1.76-fold in both scrambled siRNA and empty vector conditions, respectively (**Figure 4E**). Knockdown of Nostrill in uninfected cells resulted in a significant 0.71-fold decrease in Irf9 expression, while knockdown of Nostrill in *C. parvum*-infected cells resulted in Irf9 expression not significantly different from uninfected scrambled siRNA controls, however, this was not significantly different from scrambled siRNA *C. parvum*-infected cells (**Figure 4E**). Overexpression of Nostrill had no significant effect on Irf9 expression compared to empty vector controls in uninfected of *C. parvum*-infected cells (**Figure 4E**). Irf7 expression was significantly induced by *C. parvum* exposure to 2.14-fold and 2.78-fold in both scrambled siRNA and empty vector conditions, respectively (**Figure 4F**). Knockdown of Nostrill in uninfected cells resulted in significantly decreased expression of Irf7 to 0.67-fold of uninfected scrambled control. Significant Irf7 induction (1.41-fold) was observed in *C. parvum* infected cells with Nostrill knockdown, however, there was a significant reduction in Irf7 expression in the siRNA-Nostrill *C. parvum* infected cells compared to siRNA-Control *C. parvum* infected cells (**Figure 4F**). Conversely, Nostrill overexpression resulted in significantly increased Irf7 induction in *C. parvum* infected cells (**Figure 4F**). Interestingly, Nostrill overexpression alone in uninfected cells demonstrated a significant 2.09-fold increase in Irf7 mRNA expression compared to uninfected empty vector controls (**Figure 4F**). The above data suggest Nostrill induction is involved in the transcriptional regulation of Irf7 *in trans* in intestinal epithelial cells following *C. parvum* infection.

### Irf7 Induction Affects *C. parvum* Infection Burden and Is NF-κB Dependent in Intestinal Epithelial Cells

**Figure 5:**
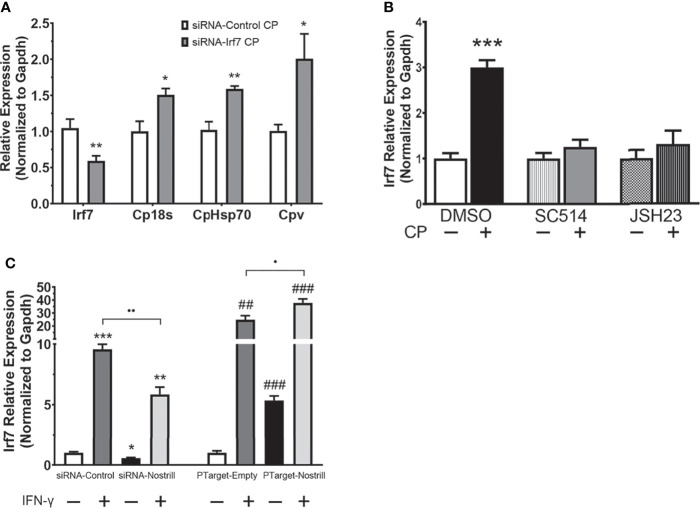
Irf7 induction by *C*. *parvum* infection contributes to anti-parasitic defense. **(A)** Impact of Irf7 induction on *C*. *parvum* infection burden *in vitro*. IEC4.1 cells were transfected with control scrambled siRNA (siRNA-Control) or a siRNA targeting Irf7 (siRNA-Irf7). Cells were then infected with *C*. *parvum* for 24 h. *C. parvum* infection burden was quantified *via* qRT-PCR. Data represent means ± SEM from three independent experiments. Gapdh was used as a reference gene for normalization. *p < 0.05 and **p < 0.01 vs siRNA-Control + CP. **(B)** *C*. *parvum*-induced Irf7 expression is NF-κB-dependent in intestinal epithelial cells. IEC4.1 cells were pre-treated with the NF-кB inhibitors SC-514 (100 μM) or JSH-23 (30 μM) 1 h prior to *C*. *parvum* infection for 24 h. DMSO was used for as a negative control. Expression was quantified *via* qRT-PCR. Data represent means ± SEM from three independent experiments. Gapdh was used as a reference gene for normalization. ***p < 0.001 vs control. **(C)** Nostrill impacts IFN-γ-mediated Irf7 expression. For knockdown, IEC4.1 cells were transfected with control scrambled siRNA (siRNA-Control) or a siRNA specific to Nostrill (siRNA-Nostrill). For Nostrill overexpression, IEC4.1 cells were transfected with Nostrill overexpression plasmid (PTarget-Nostrill) or empty vector control (PTarget-Empty). Cells were then stimulated with IFN-γ (20 ng/ml) for 24 h. Expression level of Irf7 was quantified by qRT-PCR. Gapdh was used as a reference gene for normalization. Data represent means ± SEM from three independent experiments. *p < 0.05, **p < 0.01, and ***p < 0.001 vs unstimulated siRNA-Control. ^##^p < 0.01 and ^###^p < 0.001 vs unstimulated PTarget-Empty. •p < 0.05 and ••p < 0.01 between indicated groups.

To better understand whether transcriptional regulation of Irf7 by Nostrill may be contributing to anti-*C. parvum* defense in intestinal epithelial cells, we used RNAi to knockdown Irf7 and examine the effect on parasite burden. Successful knockdown of Irf7 by the targeting siRNA is demonstrated by a significant 0.59-fold reduction in expression of Irf7 mRNA (**Figure 5A**). Knockdown of Irf7 resulted in a significant increase in *C. parvum* infection burden compared to infected scrambled siRNA controls (**Figure 5A**). Given the importance of NF-κB signaling in initiation of epithelial defense against *C. parvum*, and the presence of putative NF-κB binding sites in the promoter region of Irf7 identified by database search using TFBIND (https://tfbind.hgc.jp/), we asked whether induction of Irf7 in response to *C. parvum* infection is NF-κB dependent. Irf7 expression significantly increased 3.00-fold after *C. parvum* infection in control cells (**Figure 5B**). The use of NF-κB inhibitors SC-514 (100 µM) and JSH-23 (30 µM) resulted in the absence of *C. parvum*-induced Irf7 upregulation (**Figure 5B**), demonstrating that Irf7 is dependent on NF-κB signaling for its induction in intestinal epithelial cells in response to *C. parvum*.

To determine whether the transcriptional regulation of Irf7 by Nostrill is specific to *C. parvum* infection or is potentially present broadly in response to inflammatory stimuli, we investigated the impact of Nostrill knockdown or overexpression on Irf7 expression in response to stimulation of intestinal epithelial cells with IFN-γ. Successful knockdown or overexpression of Nostrill was observed (**Figure S2**). Stimulation with IFN-γ resulted in a more robust induction of Irf7 when compared with *C. parvum* infection in **Figure 4F**, but similar trends were observed (**Figure 5C**). Stimulation with IFN-γ resulted in a significant 9.57-fold or 24.88-fold increase in Irf7 expression compared to control cells in the scrambled siRNA or empty vector conditions, respectively (**Figure 5C**). Knockdown of Nostrill in unstimulated cells demonstrated a significant 0.56-fold decrease in Irf7 expression compared to unstimulated siRNA-Control. Knockdown of Nostrill in IFN-γ-stimulated cells resulted in a significant decrease in levels of Irf7 mRNA compared to IFN-γ-stimulated siRNA-Control cells (**Figure 5C**). Conversely, overexpression of Nostrill in unstimulated cells showed a significant 5.35-fold increase in Irf7 expression compared to unstimulated empty vector control (**Figure 5C**). Overexpression of Nostrill enhanced the IFN-γ-induced Irf7 upregulation and demonstrated a synergistic effect with a significant increase in Irf7 when compared to IFN-γ-stimulated PTarget-Empty cells (**Figure 5C**). Taken together, these data suggest that NF-κB dependent Irf7 induction participates in epithelial defense against *C. parvum* and that the transcriptional regulation of Irf7 by Nostrill is not restricted to *C. parvum* infection.

### Nostrill Promotes Irf7 Transcription Through Chromatin Modifications Associated With the NF-κB p65 Subunit

**Figure 6:**
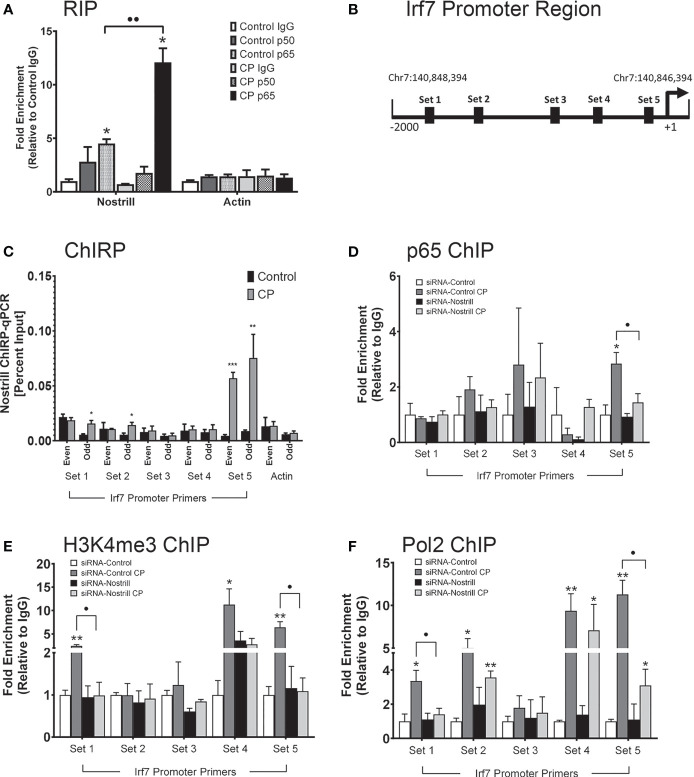
Interaction between Nostrill and NF-κB p65 and the impact on Nostrill induction on transcriptional control of Irf7. **(A)** Physical interaction between Nostrill and NF-κB p65 in intestinal epithelial cells. IEC4.1 cells were infected with *C*. *parvum* for 24 h followed by RNA immunoprecipitation (RIP) analysis using anti-p65, anti-p50, or normal IgG. Prescence of Nostrill was evaluated using qRT-PCR and the fold-enrichment method. *p < 0.05 vs control IgG. ••p < 0.01 between indicated groups. **(B)** Diagram of primer sets along *Irf7* promoter. **(C)** Recruitment of Nostrill to the *Irf7* promoter following *C*. *parvum* infection. IEC4.1 cells were infected with *C. parvum* for 24 h, followed by ChIRP analysis using two pools of probes specific to Nostrill and the PCR primer sets as designed. *p < 0.05, **p < 0.01, and ***p < 0.001 vs control. **(D–F)** Impact of Nostrill on NF-κB p65 and RNA polymerase II recruitment to the *Irf7* promoter and associated activating histone modifications following *C. parvum* infection. IEC4.1 cells were transfected with the Nostrill siRNA or control scrambled siRNA for 24 h, then infected with *C*. *parvum* for 24 h, followed by ChIP analysis using **(D)** anti-p65, **(E)** anti-H3K4me3, or **(F)** anti-Pol2 and the PCR primer sets as designed. *p < 0.05, **p < 0.01, and ***p < 0.001 vs uninfected siRNA-Control. •p < 0.05 between indicated groups. Data represent means ± SEM from three independent experiments.

Given that Nostrill has been previously found to physically associate with NF-κB p65 (19) and that Irf7 expression in response to *C. parvum* infection was observed to be NF-κB dependent, we sought to further explore whether an interaction between NF-κB and Nostrill may be present in the regulation of *Irf7* gene transactivation in the intestinal epithelium after *C. parvum* infection. We began by investigating potential physical interactions between Nostrill and the NF-κB subunits p50 and p65 through RIP assays. A significant amount of Nostrill was detected in the anti-p65 immunoprecipitates from both uninfected and *C. parvum*-infected cells (**Figure 6A**). Interestingly, there was a significant 2.68-fold increase in Nostrill detected in anti-p65 *C. parvum*-infected immunoprecipitates compared to uninfected cells (**Figure 6A**). An increase of Nostrill in anti-p50 immunoprecipitates was seen in both infected and uninfected cells, however this increase was not significant. No significant differences in uninfected or infected cells were observed in the Actin controls using anti-p50 or anti-p65 compared to non-specific IgG. ChIRP was performed next to determine whether Nostrill may occupy genomic sites in the *Irf7* gene promoter and potentially serve as a “guide” to enhance the binding of NF-κB p65. Two pools of biotinylated tiling oligonucleotide probes specific to Nostrill were used to purify chromatin fragments which were identified *via* PCR with five sets of primers in the *Irf7* promoter region (**Figure 6B**). After *C. parvum* infection, significant increases in Nostrill occupancy were detected in sets 1, 2, and 5 (**Figure 6C**). However, only set 5 showed a significant increase in Nostrill occupancy in both the even and odd pools of probes, providing stronger evidence for a true interaction between Nostrill and the *Irf7* promoter region at this site (**Figure 6C**). No significant difference in the Actin control gene was observed in *C. parvum*-infected cells compared with uninfected cells.

ChIP was next used to examine coordinated occupancy of NF-κB p65 at the *Irf7* promoter region. There was a 2.84-fold significant increase in the occupancy of NF-κB p65 at set 5 of the *Irf7* promoter region after *C. parvum* infection compared to uninfected scrambled siRNA controls (**Figure 6D**). Knockdown of Nostrill significantly reduced the *C. parvum*-induced NF-κB p65 occupancy at the set 5 region of the *Irf7* gene promoter, suggesting that Nostrill contributes to the occupancy of NF-κB p65 at the *Irf7* promoter (**Figure 6D**). Given that histone modifications are necessary for gene transcription, we queried whether Nostrill could impact H3K4 trimethylation, an activating histone modification. Significant increases in H3K4me3 were detected at the *Irf7* gene promoter at PCR primer sets 1, 4, and 5 after *C. parvum* infection in scrambled siRNA samples (**Figure 6E**). Knockdown of Nostrill significantly reduced H3K4me3 levels at set 1 and 5 after *C. parvum* exposure compared to infected scrambled siRNA controls (**Figure 6E**). Lastly, we performed ChIP to investigate the role of Nostrill in occupancy of RNA polymerase II (Pol2) at the *Irf7* promoter region after *C. parvum* infection. Significant increases in Pol2 occupancy at primer sets 1, 2, 4, and 5 were observed in scrambled siRNA controls after *C. parvum* infection (**Figure 6F**). Knockdown of Nostrill significantly reduced Pol2 occupancy at sites 1 and 5 in *C. parvum*-infected cells compared to scrambled siRNA controls (**Figure 6F**). Taken together, the above data suggest Nostrill physically interacts with NF-κB p65 and the *Irf7* gene promoter at the set 5 region to participate in the induction of Irf7 transcription in the intestinal epithelium in response to *C. parvum* infection.

### Identification of a NF-κB Dependent Putative Human Ortholog of Nostrill Impacts *C. parvum* Infection Burden

**Figure 7:**
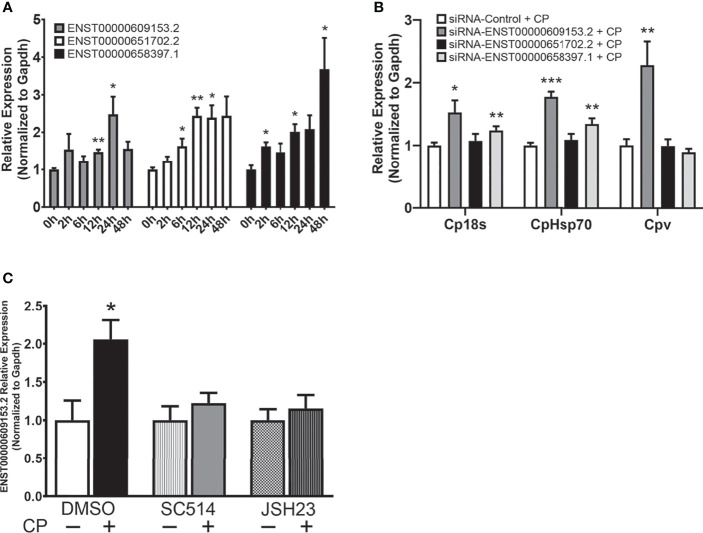
A putative NF-κB-dependent human ortholog of Nostrill contributes to anti-*C. parvum* defense. **(A)** Induction of positionally conserved human lncRNAs in response to *C*. *parvum* infection in cultured intestinal epithelial cells. HCT-8 cells were infected with *C*. *parvum* for 2-48 h and lncRNA induction was measured *via* qRT-PCR. **(B)** Impact of positionally conserved lncRNA induction on *C*. *parvum* infection burden *in vitro*. HCT-8 cells were transfected with a control scrambled siRNA (siRNA-Control) or a siRNA specific to each of the three identified positionally conserved lncRNAs then infected with *C. parvum* for 24 h. *C. parvum* infection burden was quantified *via* qRT-PCR. **(C)** *C*. *parvum*-induced ENST00000609153.2 expression is NF-κB-dependent in intestinal epithelial cells. HCT-8 cells were pre-treated with the NF-кB inhibitors SC-514 (100 μM) or JSH-23 (30 μM) 1 h prior to *C*. *parvum* infection for 24 h. DMSO was used for as a negative control. Gapdh was used as a reference gene for normalization. Data represent means ± SEM from three independent experiments. *p < 0.05, **p < 0.01, and ***p < 0.001 vs control.

LncRNAs often display low primary sequence conservation between species (36, 37), however, lncRNAs have been observed to be expressed from syntenic coding gene loci and it has been suggested this positional conservation may indicate functional commonality (38, 39). We identified 3 positionally conserved human lncRNAs localized by the *Sap30* and *Hmgb2* gene loci, ENST00000609153.2, ENST00000651702.2, and ENST00000658397.1 (**Figure S3**). Interestingly, upregulation of all three positionally conserved human lncRNAs was observed in cultured human epithelial HCT-8 cells following exposure to *C. parvum* at varying timepoints during infection (**Figure 7A**). Induction of positionally conserved orthologs was also observed in the FHs 74 Int human small intestine epithelial cell line after *C. parvum* infection and LPS stimulation (**Supplementary Figure 4**). To determine the potential of these positionally conserved lncRNAs in anti-*C. parvum* defense, we knocked down their expression utilizing the RNAi approach and measured the impact on parasite burden, similar to the experiments in **Figure 3**. Successful knockdown compared to a scrambled siRNA was confirmed (**Supplementary Figure 2**). Knockdown of both ENST00000609153.2 and ENST00000651702.2 significantly increased parasite burden as measured by Cp18s and CpHsp70 compared to scrambled siRNA control (**Figure 7B**). However, only knockdown of ENST00000609153.2 significantly impacted the infection burden as measured by Cpv, the viral symbiont carried by *C. parvum* (**Figure 7B**). Knockdown of ENST00000658397.1 had no impact on *C. parvum* infection burden. We next sought to characterize the upstream regulation of ENST00000609153.2 induction and potential similarities to Nostrill by performing experiments with inhibition of the NF-κB signaling pathway. NF-κB inhibition by both SC-514 (100 µM)and JSH-23 (30 µM) abolished the induction of ENST00000609153.2 by *C. parvum* infection observed in DMSO control samples (**Figure 7C**). Together, these preliminary results suggest ENST00000609153.2 may function as a putative human ortholog of Nostrill, whose induction is orchestrated through NF-κB signaling and contributes to epithelial defense against *C. parvum*. Further studies are needed to evaluate the underlying molecular mechanism of ENST00000609153.2 in anti-*C. parvum* defense.

## Discussion

LncRNAs are increasingly recognized as critical regulatory molecules with roles in diverse biological processes, including inflammation and host defense (40–42). Prior studies have revealed that lncRNAs, such as NR_033736, NR_045064, and XR_001779380 (16–18), participate in the epithelial antimicrobial defense response against *C. parvum*, and thus represent potential novel therapeutic targets to bolster intestinal epithelial anti-*C. parvum* defense as the current FDA approved treatments are only partially effective (43). Here, we build upon a growing body of evidence for the importance of lncRNAs in intestinal epithelial defense against *C. parvum* to report that the lncRNA Nostrill, transcribed from the *2500002B13Rik* gene locus, is significantly upregulated *via* the NF-κB signaling pathway following *C. parvum* infection.

While the expression of many lncRNAs appear to be cell-type specific, we previously identified the induction of Nostrill and its function in modulating iNOS expression in microglia and subsequent microglial-mediated neurotoxicity in response to LPS stimulation (19). LPS is a potent TLR4 agonist that strongly induces downstream NF-κB signaling, while *C. parvum* activates NF-κB signaling through TLR2 and TLR4 (44, 45). Given that NF-κB signaling plays a critical role in response to LPS stimulation and *C. parvum* infection, it isn’t surprising that the induction of Nostrill is regulated in part by NF-κB in both studies, as the NF-κB inhibitors JSH-23 and SC-514 significantly reduced *C. parvum*- or LPS-mediated induction of Nostrill (19). Thus, Nostrill may represent a broadly reactive regulator of the inflammatory response that is induced by NF-κB signaling and future studies investigating its role in response to other stimuli may be warranted. Here, we observed significant Nostrill upregulation at 24 h post-infection *in vitro, ex vivo*, and *in vivo*. However, by 48 h the expression of Nostrill returned to basal levels in *ex vivo and in vivo* model systems, while Nostrill expression was decreasing *in vitro*. Tight temporal control of Nostrill expression was previously reported (19), and additional studies are necessary to understand the factors and mechanisms regulating the temporal expression of Nostrill.

One major finding of this study is that Nostrill is required for optimal intestinal epithelial defense against *C. parvum* infection. We observed that loss-of- or gain-of-function of Nostrill significantly impacted the parasitic burden of *C. parvum* in intestinal epithelial cells utilizing *in vitro* and *ex vivo* 2D monolayer model systems. Future studies are required to examine the role of Nostrill in defense against *C. parvum in vivo* and in 3D enteroids where cellular organization and architecture differs. Knowing that lncRNAs can regulate target gene transcription both *in cis* and *in trans*, we examined the potential of Nostrill as a cis-acting transcriptional regulator by measuring the effect of Nostrill modulation on expression of nearby genes *Hmgb2* and *Sap30*. Alteration of Nostrill expression did not significantly impact the transcription of Hmgb2, while knockdown of Nostrill further significantly reduced *C. parvum*-induced downregulation of Sap30 expression. However, the overexpression of Nostrill did not significantly impact the expression of the *Sap30* gene locus. Given that knockdown of Nostrill further reduced Sap30 expression following *C. parvum* infection and overexpression of Nostrill ameliorated *C. parvum*-induced downregulation of Sap30, this provides weak evidence that Nostrill may regulate Sap30 expression and further mechanistic studies are necessary to explore this possibility. We investigated the Nostrill-mediated transactivation of iNOS, as iNOS inhibition has been shown to delay *C. parvum* clearance *in vivo* through direct effects of iNOS and NO which were not due to reduced turnover of cells in the intestinal epithelium (46). Suggested mechanisms of antiparasitic defense due to iNOS include generation of peroxynitrite at sites of *C. parvum* infection as treatment with peroxynitrite scavengers exacerbated *C. parvum* infection, in addition to inhibition of *C. parvum* excystation and viability by iNOS (47, 48). Despite the role of Nostrill in regulating iNOS expression in the microglial inflammatory response, modulation of Nostrill in the intestinal epithelium didn’t significantly affect the expression of iNOS in response to *C. parvum* infection, demonstrating that Nostrill may possess cell-type or stimuli specific regulatory capabilities. LncRNAs have been found to directly impact the catalytic activity of enzymes (49), and investigation into nitric oxide production following Nostrill manipulation may help determine whether Nostrill impacts the catalytic activity of iNOS without affecting its transcription.

It is well appreciated that IFN signaling plays a critical role in antiparasitic defense against *C. parvum*. Treatment with type I IFNs (IFN-α/β) or the type II IFN (IFN-γ) prior to *Cryptosporidium* infection has been shown to enhance resistance to infection (12, 13), while a novel role for type III IFN (IFN-λ) in anti-*C. parvum* defense was recently demonstrated (50). In mammalian cells, the nine members of the interferon regulatory factor (IRF) family regulate the interferon response, with IRF7 being implicated as the master regulator of type I IFN signaling (51, 52). Following activation of IRF7 by downstream signaling pathways of pattern recognition receptors, including members of the TLR family, IRF7 acts to control the induction of >300 IFN-inducible genes (52). Genome-wide expression analyses have demonstrated that modulation of a single lncRNA can impact the expression of many downstream genes, bolstering their importance as critical regulators of biological functions. Additionally, recent microarray analysis of the intestinal epithelium at the peak of *Cryptosporidium* infection demonstrated significant upregulation of many interferon-stimulated genes (50). For example, we found that the lncRNA NR_033736 negatively regulates a subset of type I IFN-controlled genes, including *Usp18*, *Igtp*, *Iigp*, *Ifit1*, *Ifi44*, *Iigp1*, *Oas2*, and *Max2* (16). Given the data supporting the interferon response in anti-*C. parvum* defense, we focused our analysis on regulators of IFN signaling. Interestingly, we found that Nostrill regulates Irf7 expression in response to *C. parvum* infection in the intestinal epithelium. Knockdown of Nostrill significantly reduced the Irf7 expression in both uninfected and *C. parvum* infected cells, while overexpression of Nostrill significantly enhanced Irf7 expression in both uninfected and *C. parvum* infected cells. Additionally, we saw that knockdown of Irf7 significantly increased the parasitic burden of *C. parvum*, providing further evidence for its critical role in anti-*C. parvum* defense. Given the importance of type I IFN signaling in defense against *Cryptosporidium* infection, further studies regarding Irf7 and the role of Nostrill in Irf7 regulation as a potential antiparasitic therapeutic may be warranted. However, we cannot exclude the possibility here that Nostrill impacts or regulates the transcription of other defense genes in the anti-*Cryptosporidium* response, and genome-wide expression analyses are required to further characterize the complete role of Nostrill in the intestinal epithelium in response to *C. parvum* infection.

Our previous study demonstrated that Nostrill physically interacts with the NF-κB p65 subunit which possesses the transactivation domain necessary for transcriptional activity. In addition, because *Irf7* contains multiple canonical NF-κB p65 binding sites in its promoter region and we observed that induction of Irf7 in response to *C. parvum* infection was dependent on NF-κB signaling, we evaluated whether Nostrill may interact with NF-κB p65 to impact *Irf7* gene transcription. In the current study, RIP analysis revealed an interaction between Nostrill and NF-κB p65 in both uninfected and *C. parvum* infected cells, with a significant increase observed between Nostrill and NF-κB p65 after *C. parvum* infection. Interaction between NF-κB p50 and Nostrill was elevated, although not significantly, compared to IgG controls. Knockdown of Nostrill significantly reduced association of NF-κB p65 with the *Irf7* promoter at primer set 5 after *C. parvum* infection as observed *via* ChIP, and additionally significantly reduced presence of the activating histone modification H3K4me3 and presence of RNA polymerase II at the same site. Recruitment of Nostrill to the set 5 region of the *Irf7* promoter was confirmed by ChIRP. Together, this data supports that Nostrill may function as a “guide” to facilitate recruitment of NF-κB p65 to the *Irf7* gene promoter to promote Irf7 mRNA expression. A recent study identified Nostrill as a chromatin architectural protein (CAP) associated lncRNA, which may be involved in the role of CAPs in chromatin organization and interactions between promoters and enhancers, however the functional mechanism of Nostrill was not further elucidated (53). Further studies are required to elucidate the role of Nostrill as a “scaffold” between NF-κB p65, Nostrill, and other potential proteins, including RNA polymerase II and CAPs.

Poor lncRNA sequence conservation has been noted between species (36, 37), however, large studies have observed positional conservation (38). Here, we identified three positionally conserved lncRNAs in humans that were induced in response to *C. parvum* infection. One of these lncRNAs, ENST00000609153.2, is NF-κB-dependent and significantly impacts infection burden of *C. parvum*, similar to Nostrill. Further studies are needed to evaluate the molecular mechanism of ENST00000609153.2 as it contributes to anti-*C. parvum* defense, and whether delivery of ENST00000609153.2 can limit the parasitic burden of *C. parvum* in human cells.

In summary, our data indicate that the NF-κB-dependent lncRNA Nostrill contributes to intestinal epithelial defense against *C. parvum* through interactions with NF-κB p65 and the *Irf7* gene promoter. Identification of positionally conserved human orthologs, including the lncRNA ENST00000609153.2, promote epithelial defense against *C. parvum* through an unexplored mechanism, which may or may not be similar to Nostrill. These *in vitro* and *ex vivo* studies are the first step in evaluating the therapeutic potential of Nostrill in augmenting current treatment strategies for *C. parvum* infection. Future studies examining the role of Nostrill *in vivo* and the functional mechanism of its putative human orthologs are required to further our understanding of novel targets for RNA therapeutics.

## Data Availability Statement

The original contributions presented in the study are included in the article/**Supplementary Material**. Further inquiries can be directed to the corresponding author.

## Ethics Statement

The animal study was reviewed and approved by Creighton University IACUC Committee.

## Author Contributions

NM and X-MC designed experiments and wrote the manuscript. NM, SD, A-YG, ML, YW, OB, AK, and EW performed experiments. NM, SD, A-YG, ML, YW, OB, AK, EW, AS, and X-MC performed data analysis. A-YG, AS, and X-MC directed and supervised the study. All authors contributed to the article and approved the submitted version.

## Funding

This work was supported by funding from the National Institutes of Health (AI116323, AI136877, AI141325, and AI156370) to X-MC. The project described was also supported by grants P20 GM103427 and P20 GM139762-01 from the National Center for Research Resources.

## Author Disclaimer

The content is solely the responsibility of the authors and does not necessarily represent the official views of the National Institutes of Health.

## Conflict of Interest

The authors declare that the research was conducted in the absence of any commercial or financial relationships that could be construed as a potential conflict of interest.

## Publisher’s Note

All claims expressed in this article are solely those of the authors and do not necessarily represent those of their affiliated organizations, or those of the publisher, the editors and the reviewers. Any product that may be evaluated in this article, or claim that may be made by its manufacturer, is not guaranteed or endorsed by the publisher.
