# Supplementary material for: The Long Non-Coding RNA Nostrill Regulates Transcription of Irf7 Through Interaction With NF-κB p65 to Enhance Intestinal Epithelial Defense Against Cryptosporidium parvum
Source: Front Immunol. 2022 Apr 7;13:863957. doi: 10.3389/fimmu.2022.863957 (PMC9021721; doi:10.3389/fimmu.2022.863957)
Supplement: Supplementary file 1 [file Presentation_1.pptx]

## Slide 1
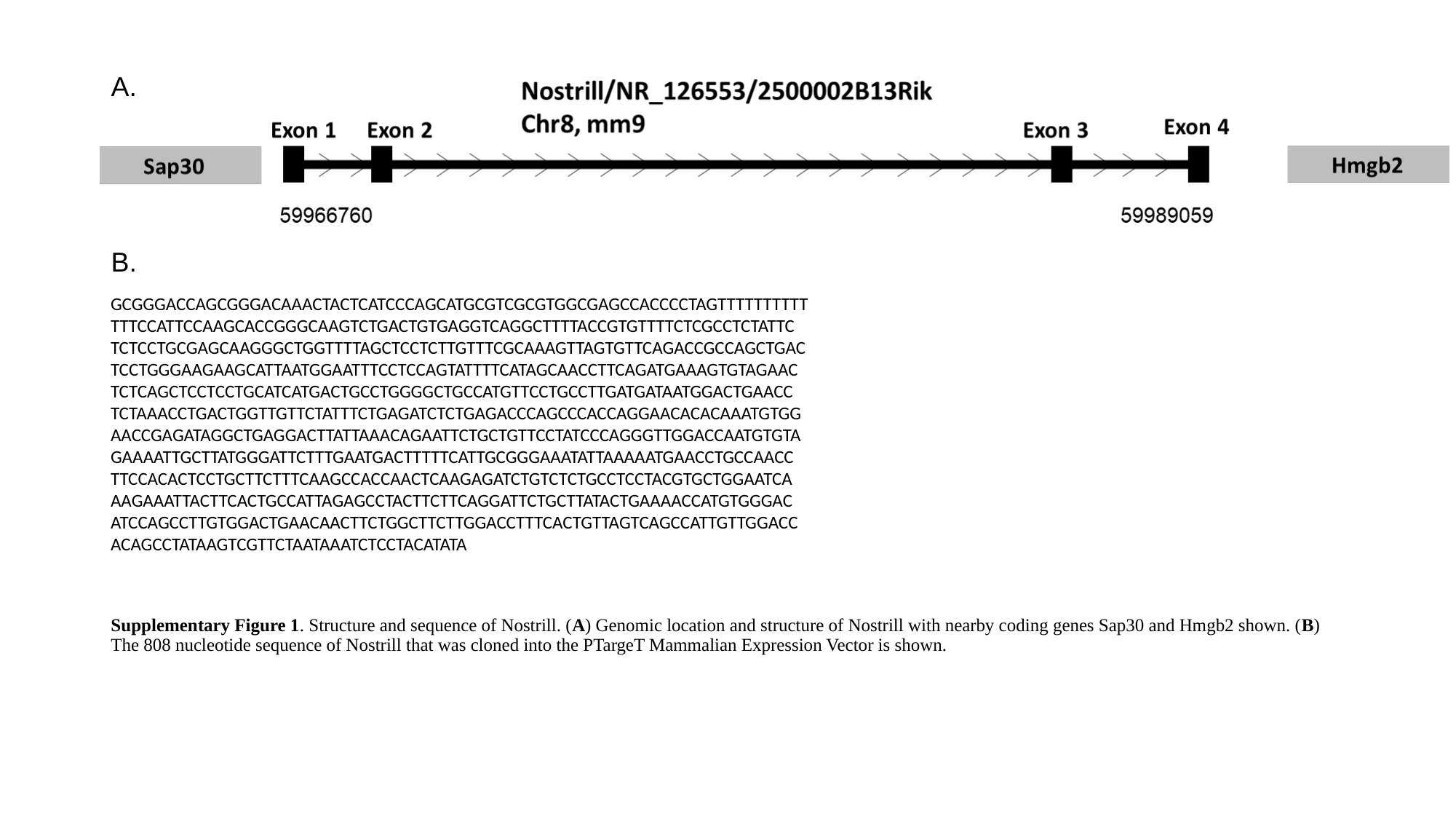

A.
B.
GCGGGACCAGCGGGACAAACTACTCATCCCAGCATGCGTCGCGTGGCGAGCCACCCCTAGTTTTTTTTTT
TTTCCATTCCAAGCACCGGGCAAGTCTGACTGTGAGGTCAGGCTTTTACCGTGTTTTCTCGCCTCTATTC
TCTCCTGCGAGCAAGGGCTGGTTTTAGCTCCTCTTGTTTCGCAAAGTTAGTGTTCAGACCGCCAGCTGAC
TCCTGGGAAGAAGCATTAATGGAATTTCCTCCAGTATTTTCATAGCAACCTTCAGATGAAAGTGTAGAAC
TCTCAGCTCCTCCTGCATCATGACTGCCTGGGGCTGCCATGTTCCTGCCTTGATGATAATGGACTGAACC
TCTAAACCTGACTGGTTGTTCTATTTCTGAGATCTCTGAGACCCAGCCCACCAGGAACACACAAATGTGG
AACCGAGATAGGCTGAGGACTTATTAAACAGAATTCTGCTGTTCCTATCCCAGGGTTGGACCAATGTGTA
GAAAATTGCTTATGGGATTCTTTGAATGACTTTTTCATTGCGGGAAATATTAAAAATGAACCTGCCAACC
TTCCACACTCCTGCTTCTTTCAAGCCACCAACTCAAGAGATCTGTCTCTGCCTCCTACGTGCTGGAATCA
AAGAAATTACTTCACTGCCATTAGAGCCTACTTCTTCAGGATTCTGCTTATACTGAAAACCATGTGGGAC
ATCCAGCCTTGTGGACTGAACAACTTCTGGCTTCTTGGACCTTTCACTGTTAGTCAGCCATTGTTGGACC
ACAGCCTATAAGTCGTTCTAATAAATCTCCTACATATA
Supplementary Figure 1. Structure and sequence of Nostrill. (A) Genomic location and structure of Nostrill with nearby coding genes Sap30 and Hmgb2 shown. (B) The 808 nucleotide sequence of Nostrill that was cloned into the PTargeT Mammalian Expression Vector is shown.

## Slide 2
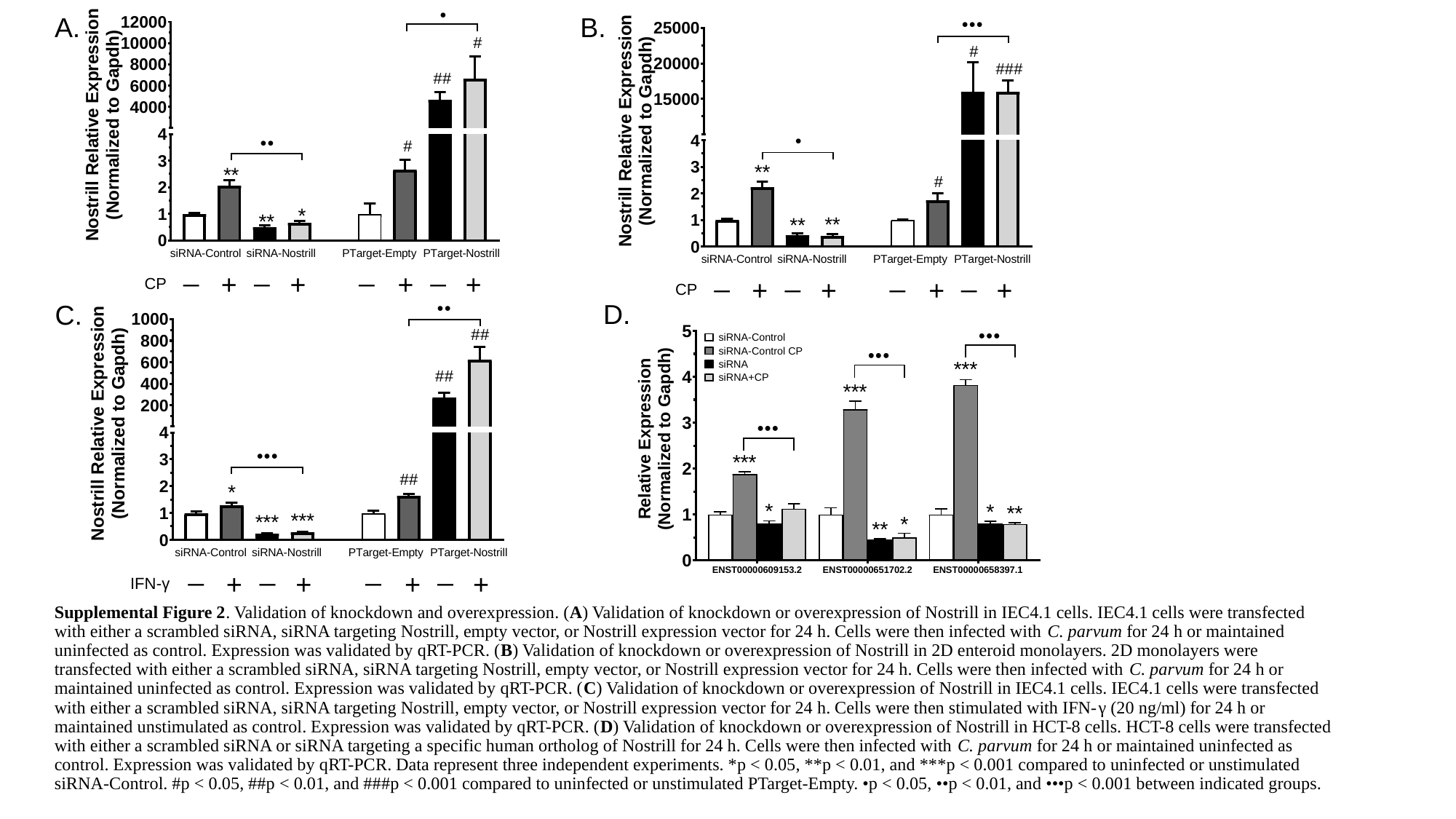

A.
B.
D.
C.
Supplemental Figure 2. Validation of knockdown and overexpression. (A) Validation of knockdown or overexpression of Nostrill in IEC4.1 cells. IEC4.1 cells were transfected with either a scrambled siRNA, siRNA targeting Nostrill, empty vector, or Nostrill expression vector for 24 h. Cells were then infected with C. parvum for 24 h or maintained uninfected as control. Expression was validated by qRT-PCR. (B) Validation of knockdown or overexpression of Nostrill in 2D enteroid monolayers. 2D monolayers were transfected with either a scrambled siRNA, siRNA targeting Nostrill, empty vector, or Nostrill expression vector for 24 h. Cells were then infected with C. parvum for 24 h or maintained uninfected as control. Expression was validated by qRT-PCR. (C) Validation of knockdown or overexpression of Nostrill in IEC4.1 cells. IEC4.1 cells were transfected with either a scrambled siRNA, siRNA targeting Nostrill, empty vector, or Nostrill expression vector for 24 h. Cells were then stimulated with IFN-γ (20 ng/ml) for 24 h or maintained unstimulated as control. Expression was validated by qRT-PCR. (D) Validation of knockdown or overexpression of Nostrill in HCT-8 cells. HCT-8 cells were transfected with either a scrambled siRNA or siRNA targeting a specific human ortholog of Nostrill for 24 h. Cells were then infected with C. parvum for 24 h or maintained uninfected as control. Expression was validated by qRT-PCR. Data represent three independent experiments. *p < 0.05, **p < 0.01, and ***p < 0.001 compared to uninfected or unstimulated siRNA-Control. #p < 0.05, ##p < 0.01, and ###p < 0.001 compared to uninfected or unstimulated PTarget-Empty. •p < 0.05, ••p < 0.01, and •••p < 0.001 between indicated groups.

## Slide 3
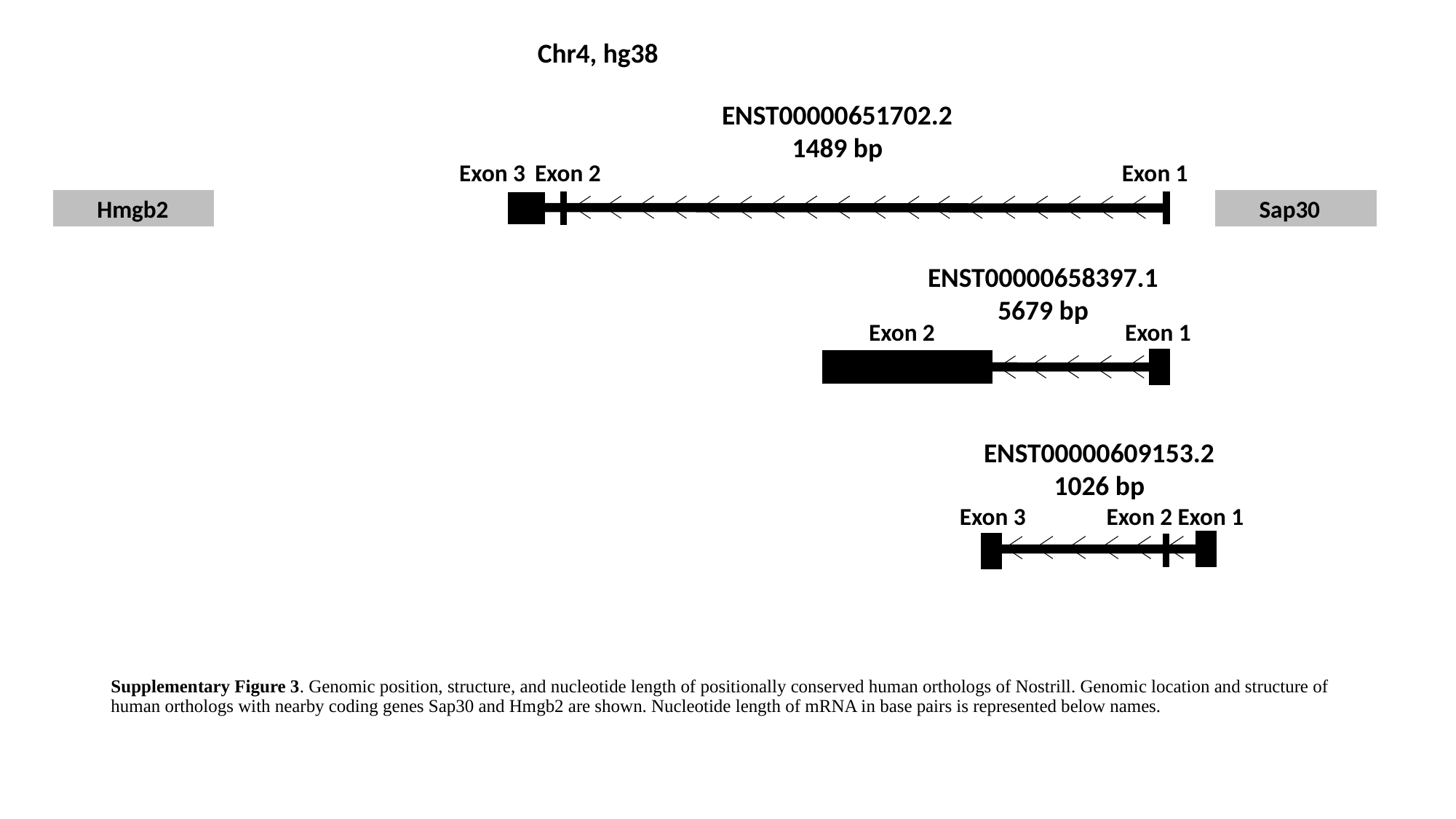

Chr4, hg38
ENST00000651702.2
1489 bp
Exon 3
Exon 2
Exon 1
Hmgb2
Sap30
ENST00000658397.1
5679 bp
Exon 2
Exon 1
ENST00000609153.2
1026 bp
Exon 3
Exon 2
Exon 1
Supplementary Figure 3. Genomic position, structure, and nucleotide length of positionally conserved human orthologs of Nostrill. Genomic location and structure of human orthologs with nearby coding genes Sap30 and Hmgb2 are shown. Nucleotide length of mRNA in base pairs is represented below names.

## Slide 4
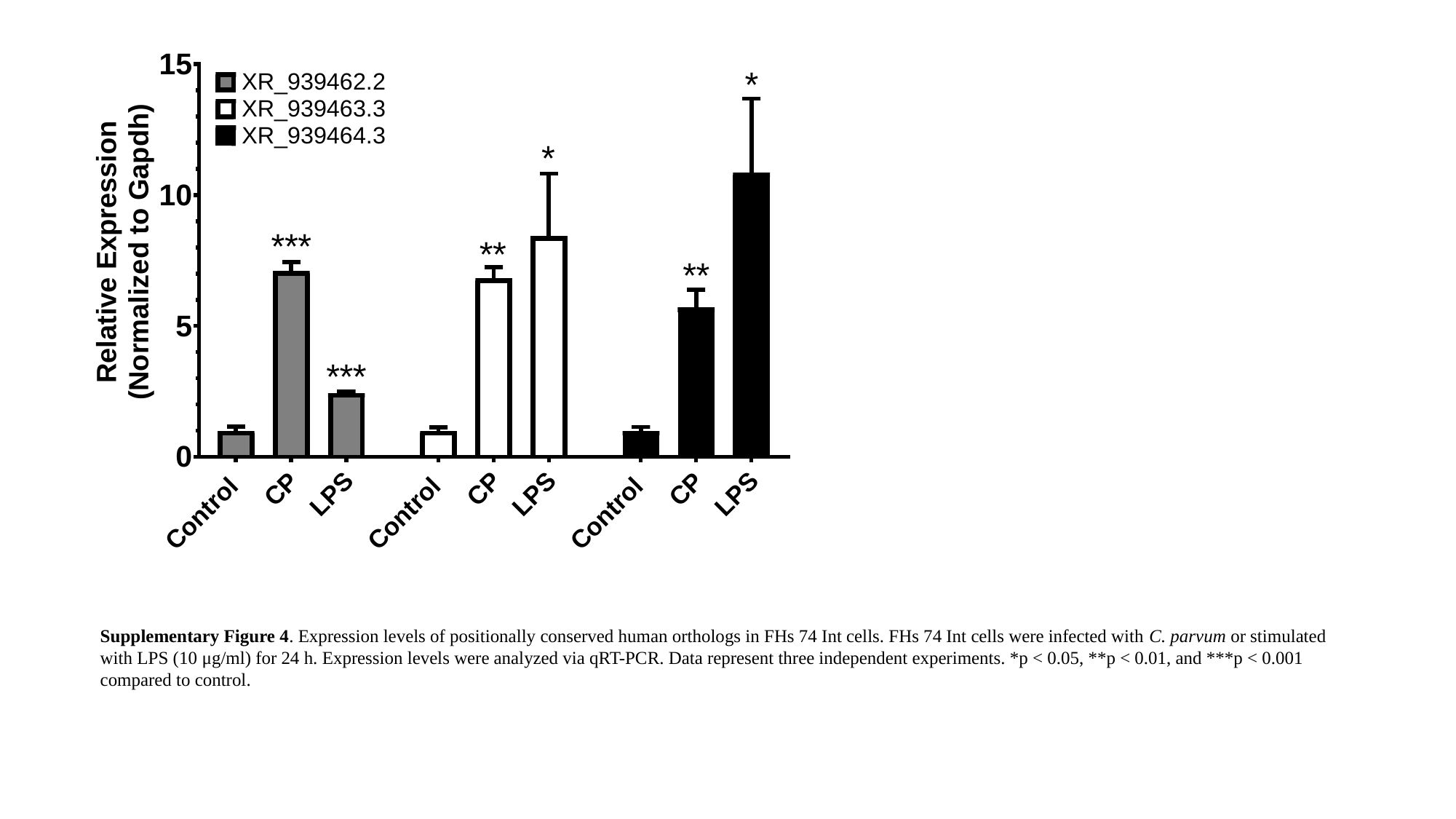

Supplementary Figure 4. Expression levels of positionally conserved human orthologs in FHs 74 Int cells. FHs 74 Int cells were infected with C. parvum or stimulated with LPS (10 μg/ml) for 24 h. Expression levels were analyzed via qRT-PCR. Data represent three independent experiments. *p < 0.05, **p < 0.01, and ***p < 0.001 compared to control.
